# Transcutaneous Electrical Nerve Stimulation in Rodent Models of Neuropathic Pain: A Meta-Analysis

**DOI:** 10.3389/fnins.2022.831413

**Published:** 2022-01-31

**Authors:** Jiapeng Huang, Chunlan Yang, Kehong Zhao, Ziqi Zhao, Yin Chen, Tingting Wang, Yun Qu

**Affiliations:** ^1^Department of Rehabilitation Medicine, West China Hospital, Sichuan University, Chengdu, China; ^2^Key Laboratory of Rehabilitation Medicine in Sichuan Province, West China Hospital, Sichuan University, Chengdu, China; ^3^Research Laboratory of Neurorehabilitation, Research Institute of Rehabilitation Medicine, West China Hospital, Sichuan University, Chengdu, China

**Keywords:** transcutaneous electrical nerve stimulation, neuropathic pain, animal studies, pain models, meta-analysis

## Abstract

Transcutaneous electrical nerve stimulation (TENS) is a non-invasive therapeutic intervention that is typically used for many years to treat chronic pain in patients who are refractory to pain medications. However, evidence of the efficacy of TENS treatment for neuropathic pain is lacking in humans. To further understand the efficacy of TENS under various intervention conditions and illuminate the current circumstance and future research directions, we systematically reviewed animal studies investigating the efficacy of TENS in relieving pain in neuropathic pain rodent models. We searched the Cochrane Library, EMBASE, MEDLINE (via PubMed), and Web of Science and identified 11 studies. Two meta-analyses were performed. The first meta-analysis showed that a single TENS treatment was capable of temporarily ameliorating neuropathic pain when compared to control groups with a significant effect (standardized mean difference: 1.54; 95% CI: 0.65, 2.42; *p* = 0.0007; *I*^2^ = 58%). Significant temporary alleviation in neuropathic pain intensity was also observed in the meta-analysis of repetitive TENS (standardized mean difference: 0.85; 95% CI: 0.31, 1.40; *p* = 0.002; *I*^2^ = 75%). Subgroup analysis showed no effect of the timing of the application of TENS (test for subgroup difference, *p* = 0.47). Leave-one-out sensitivity analyses suggested that no single study had an outsized effect on the pooled estimates, which may partly prove the robustness of these findings. Other stratified analyses were prevented by the insufficient number of included studies. Overall, current data suggest that TENS might be a promising therapy to ameliorate neuropathic pain. However, the high risk of bias in the included studies suggests that cautions must be considered when interpreting these findings and it is not reasonable to directly generalize the results obtained from animal studies to clinical practice. Future studies should pay more attention to improving the quality of study design and reporting, thereby facilitating the understanding of mechanisms underlying TENS treatment, reducing more potentially unsuccessful clinical trials, and optimizing the efficacy of TENS for people with neuropathic pain.

## Introduction

Neuropathic pain, which is caused by an injury or disease of the somatosensory system, is characterized by spontaneous pain, hyperalgesia, and allodynia and can be classified as peripheral or central neuropathic pain according to the site of injury or disease (Treede et al., 2008). It is estimated that the prevalence of neuropathic pain is 6.9% to 10% (van Hecke et al., 2014). Neuropathic pain represents an important source of chronic pain and dysfunction and causes a significant burden on people and society (Jensen et al., 2011). Therefore, the management of neuropathic pain should be important to ease its negative impact on activities of daily living and quality of life (Andrew et al., 2014; Leadley et al., 2014).

The mainstay of interventions for neuropathic pain is primarily pharmacological (Dworkin et al., 2013); however, for the large number of patients who cannot benefit from pharmacological intervention or who experience unwanted side effects, improving the ability to effectively relieve neuropathic pain with a non-pharmacological intervention such as psychological or physical treatment is crucial (Somers and Clemente, 2009; Gibson et al., 2017). Transcutaneous electrical nerve stimulation (TENS) is a non-invasive, safe, easy to administer, portable, and inexpensive technique that delivers pulsed electrical stimulation, which can be modified regarding frequency, current intensity, and duration, *via* two or more skin electrodes to stimulate underlying nerves for pain control and has an advantage of allowing patients to control their pain autonomously (Pal et al., 2020). The antinociceptive effect of TENS may involve peripheral receptors (Santos et al., 2013), spinal (Melzack and Wall, 1965; Wall and Sweet, 1967), and supraspinal mechanisms (Kalra et al., 2001; DeSantana et al., 2008, 2009). And the application of TENS is based on the pain gate theory, which proposes that the stimulation of large diameter (A-β) afferent fibers may close the pain gate and alleviate the pain (Melzack and Wall, 1965; DeSantana et al., 2008). Besides, TENS treatment has been shown to relieve pain by reducing the sensitization of dorsal horn neurons (Sabino et al., 2008), elevating levels of gamma-aminobutyric acid and glycine (Maeda et al., 2007; Somers and Clemente, 2009), and inhibiting glial activation (Matsuo et al., 2014). The two most common types of TENS treatment are high-frequency (50 or 100 Hz and above), low-intensity TENS and low-frequency (10 Hz or less), high-intensity TENS (Hurlow et al., 2012; Gibson et al., 2017). However, the proof of the efficacy of TENS for neuropathic pain is limited and the TENS parameter that would best treat neuropathic pain remains unclear. A Cochrane Review of TENS for neuropathic pain reported that they cannot confidently state whether TENS is efficacy for neuropathic control due to the low-quality evidence obtained from a small number of studies included in the meta-analysis, and the lack of clinical studies prevented further subgroup analyses, resulting in the optimal pattern of TENS remaining unknown (Gibson et al., 2017). Furthermore, studies suggested that electrical stimulation exerted an antinociceptive effect in a specific time window (Kerns and Lucchinetti, 1992; Su et al., 2018), whereas there were various inconsistencies amongst previous studies with respect to the nociceptive effect according to the timing of intervention tested (Somers and Clemente, 1998; Su et al., 2018). In contrast to human trials, animal studies are more exploratory and enable the additional design of independent variables and the control of confounders. Previous animal studies have explored the effect of TENS on neuropathic pain, but results have not been consistent (Somers and Clemente, 2009; Lin et al., 2015; Su et al., 2018). It is worth noting that systematic reviews of preclinical studies have the potential to inform future clinical trials and thereby ease translational challenges (Harman et al., 2020). Therefore, a systematic review of animal studies exploring the efficacy of TNES for neuropathic pain is important and desirable. However, no meta-analysis has assessed the antinociceptive effect of TENS in alleviating neuropathic pain.

Based on the above background, we focused on a single TENS and repetitive TENS, with the primary purpose being to assess the efficacy of TENS in relieving pain in rodent models of neuropathic pain. The second purpose was to evaluate whether the efficacy of TENS is influenced by the TENS parameters and experimental design. The third purpose was to clarify the current circumstance and future research directions of TENS.

## Materials and Methods

The present meta-analysis followed the guidelines of Preferred Reporting Items for Systematic Review and Meta-analyses (Johnson et al., 2004) (Supplementary Material 1). The protocol for this study was available online (registration number: INPLASY2021110104). No ethical approval was needed as all information was extracted from studies published previously.

### Search Strategy

Animal studies investigating the efficacy of TENS for neuropathic pain were identified by searching electronic databases, including Cochrane Library, EMBASE, MEDLINE (*via* PubMed), and Web of Science. Search terms included pain, transcutaneous electrical nerve stimulation, muridae, and keywords that were confirmed following multiple pre-searches (Supplementary Material 2).

### Criteria for Considering Studies for This Meta-Analysis

The inclusion criteria for this meta-analysis were as follows: (1) Animal studies using rodent models of neuropathic pain induced by one of the following methods: chronic constriction injury (CCI), spared never injury (SNI), spinal cord injury (SCI), spinal nerve ligation (SNL), nerve crush injury (NCI), viral infection for postherpetic neuralgia, plexus ablation, chemotherapeutics, streptozotocin administration, or central lesions (Velzen et al., 2021); (2) Rodents in experiment groups received all standard models of TENS with unlimited frequency, intensity, duration, and timing of intervention; (3) Neuropathic pain-inducing rodents in the control group should receive sham TENS or blank treatment, except usual anesthesia; (4) Studies had to provide quantitative data on pain, irrespective of the type of pain, which can be measured by a mechanical threshold, thermal threshold, or cold threshold. And pain can be expressed as an absolute value or a percentage.

Exclusive criteria were as follows: (1) studies using rodent models of inflammation pain, non-inflammation pain, or cancer pain, and those utilizing non-rodent models, humans, or *ex vivo* and *in vitro* preparations; (2) studies in which pulsed electrical stimulation was delivered percutaneously such as electroacupuncture (EA) and percutaneous electrical nerve stimulation (PENS) or in which rodents received vagus/trigeminus nerve stimulation or acupuncture points stimulation, including, but not limited to transcutaneous electrical acupoint stimulation (TEAS); (3) TENS was utilized in conjunction with another intervention; (4) studies not including an independent control group that did not receive active TENS; (5) Conference abstract, editorial, review, and non-English publications.

According to the above criteria, two reviewers independently read the titles and abstracts of the retrieved records and eliminated apparently irrelevant studies. Subsequently, the full text of the remaining studies was retrieved, and two investigators independently assessed the studies for final inclusions. In case of ambiguity, we contacted the authors to provide additional information *via* email. Discrepancies were resolved through discussion, or by consulting a third investigator.

### Data Extraction and Quality Assessment

TENS and control data about paw withdrawal thresholds expressed as an absolute value or a percentage after TENS treatment at identical time points were extracted. When quantitative data were not explicitly reported in text and supplementary materials, we extracted the data from figures using Engauge Digitizer (Huang et al., 2021). The primary outcome mechanical threshold was used in the meta-analysis if reported and available (91% of included studies), otherwise thermal threshold or cold threshold was used as an alternative. First author information, year of publication, species, strain, age, sex, weight, sample size per group, modeling methods, the protocol of TENS, parameters of TENS, the timing of TENS, type of control intervention, anesthesia used during TENS treatment, outcome measurement methods, adverse events, and so on were also extracted. Two authors independently extracted data and then discussed or consulted a third reviewer to resolve discrepancies. The authors of included studies would not be contacted to provide missing data which has not been peer-reviewed.

Two investigators independently evaluated the risk of bias of included studies utilizing the SYRCLE’s risk of bias tool for animal studies (Hooijmans et al., 2014b). High-bias risk, low-bias risk, and unclear bias risk were used to grade the included studies. We discussed, or consulted a third investigator to make final decisions.

### Data Analysis

All meta-analyses and graphical displays were conducted using RevMan 5.3 (The Cochrane Collaboration, Copenhagen, Denmark). A random-effects model was used due to the exploratory nature of animal studies and the anticipated heterogeneity. If methods of outcome measurement or forms of data expression were different among the included studies, we calculated a standardized mean difference (SMD) to summarize effects from studies in this meta-analysis; otherwise, a mean difference (MD) was used. To ensure that the results had the same directional value, we multiplied one kind of outcome by −1 if the change direction to reflect the relief degree of neuropathic pain was different. To prevent double-counting sample sizes of control animals, we split the animal number of the control group in case of studies using a single control group and multiple experimental groups. We used *I*^2^ to evaluate the heterogeneity. Where comparable data were available from at least three studies, we planned subgroup analysis in the following domains: frequency, the timing of intervention, intensity, electrode placement, species, method of modeling, the timing of outcome measurement, and anesthesia used during intervention procedures. We evaluated the robustness of the results using leave-one-out sensitivity analyses. For studies that could not be included in the meta-analysis, we performed a descriptive summary. The publication bias would be analyzed using a funnel plot in case of at least 10 studies were included in a certain subgroup; otherwise, we would not analyze the publication bias.

## Results

### Results of the Search

**FIGURE 1:**
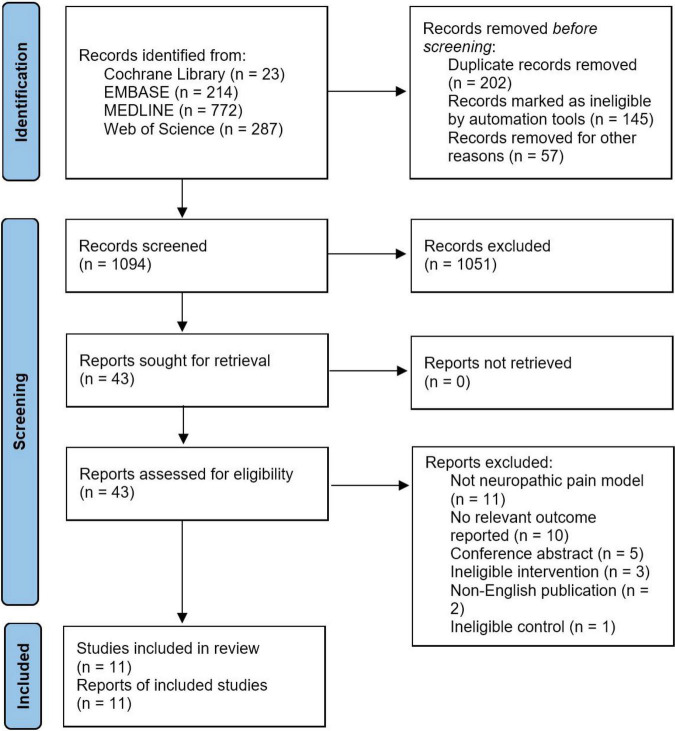
PRISMA flow diagram for search strategy and study selection.

Figure 1 shows the flow diagram for search strategy and study selection process. The literature search was conducted on November 12, 2021. We initially retrieved 1,296 potentially eligible records, of which 23 studies from Cochrane Library, 214 from EMBASE, 772 from MEDLINE, and 287 from Web of Science. We removed duplicates with 1,094 records were left for title-abstract screening, resulting in 1,051 records being discarded, mostly because of irrelevant research topics and ineligible treatment modalities such as EA. Forty-three records were remained to determine their eligibility by carefully full-text screening, followed by 32 records were excluded from this review for various reasons. As a result, a total of 11 studies were included and all of them were included in the quantitative synthesis (Somers and Clemente, 1998, 2003, 2006, 2009; Nam et al., 2001; Inoue et al., 2003; Vera-Portocarrero et al., 2013; Cho et al., 2014; Matsuo et al., 2014; Lin et al., 2015; Su et al., 2018).

### Characteristics of Included Studies

Table 1 shows the characteristics of studies included in the meta-analysis. Sample sizes of the included studies ranged from 16 to 66. Regarding the species employed in the included studies, 91% (10/11) of studies (Somers and Clemente, 1998, 2003, 2006, 2009; Nam et al., 2001; Inoue et al., 2003; Vera-Portocarrero et al., 2013; Cho et al., 2014; Lin et al., 2015; Su et al., 2018) employed Sprague Dawley rats, and the remaining one study (Matsuo et al., 2014) used ICR/JCL mice. In terms of sex and weight, male animals were used in all of the included studies and the weight of the animals varied across the included studies. For the neuropathic pain models, CCI (6/11) was the most common method for neuropathic pain modeling, followed by SNI (3/11), SNL (1/11), and NCI (1/11). With regard to the protocol of TENS, two studies (Nam et al., 2001; Cho et al., 2014) used a single session of TENS treatment and nine studies (Somers and Clemente, 1998, 2003, 2006, 2009; Inoue et al., 2003; Vera-Portocarrero et al., 2013; Matsuo et al., 2014; Lin et al., 2015; Su et al., 2018) employed repetitive TENS treatment, and of which one study (Vera-Portocarrero et al., 2013) provided the data about the effect of TENS following a single treatment. In addition, five studies (Somers and Clemente, 1998, 2006, 2009; Matsuo et al., 2014; Su et al., 2018) reported multiple separate comparisons (four in two studies, three in two studies, and two in one studies). As for the frequency of TENS, high-frequency was the most common one, which was used in seven studies (Somers and Clemente, 1998, 2003, 2006, 2009; Vera-Portocarrero et al., 2013; Cho et al., 2014; Matsuo et al., 2014; Lin et al., 2015), low-frequency was used in two studies (Nam et al., 2001; Inoue et al., 2003), and one study (Su et al., 2018) used both high-frequency and low-frequency TENS. Ipsilateral TENS was explicitly reported in seven studies (Somers and Clemente, 1998, 2003; Nam et al., 2001; Inoue et al., 2003; Cho et al., 2014; Matsuo et al., 2014; Lin et al., 2015), contralateral TNES was explicitly used in one study (Somers and Clemente, 2009), and one study compared the effect of ipsilateral and contralateral TENS (Somers and Clemente, 2006). However, two studies (Vera-Portocarrero et al., 2013; Su et al., 2018) did not explicitly which side of the body received TENS treatment. In terms of the intensity of TENS, all studies used sub-motor threshold, except one study (Nam et al., 2001) employed motor threshold and one study (Su et al., 2018) did not report on the intensity of TENS. Variations in the duration of TENS were observed, which ranged from 16.7 to 90 min. Regarding the control intervention, animals in seven studies (Somers and Clemente, 1998, 2003, 2006, 2009; Inoue et al., 2003; Lin et al., 2015; Su et al., 2018) received no TENS, and sham TENS was used in the remaining four studies (Nam et al., 2001; Vera-Portocarrero et al., 2013; Cho et al., 2014; Matsuo et al., 2014). Anesthesia was administrated during TENS treatment in all of the included studies, of which halothane (Somers and Clemente, 1998, 2003, 2006; Inoue et al., 2003) and isoflurane (Nam et al., 2001; Vera-Portocarrero et al., 2013; Lin et al., 2015; Su et al., 2018) were the most common anesthesia, followed by pentobarbital (Inoue et al., 2003). However, two studies (Cho et al., 2014; Matsuo et al., 2014) did not report on which type of anesthesia was used during TENS treatment.

**TABLE 1:** Characteristics of studies included in the meta-analysis, *K* = 11.

Study	Animals					Modeling method	Parameters of TENS					Timing of intervention	Control intervention	Anesthesia used during intervention procedures
	Species	Strain	Age (wk)		Weight (g)		Frequency (Hz)	Intensity	Duration (min)	Electrical placement	Protocol			
Cho et al., 2014	Rat	Sprague Dawley	–	Male	200–250	SNI	100	Sub-motor threshold	20	Ipsilateral	Single TENS	4 weeks after modeling	Sham TENS	Brief anesthesia
Nam et al., 2001	Rat	Sprague Dawley	–	Male	150–200	SNL	2	Motor threshold	20	Ipsilateral	Single TENS	3 days after modeling	Sham TENS	2% enflurane-O_2_ mixture
Vera-Portocarrero et al., 2013	Rat	Sprague Dawley	–	Male	250–300	SNI	100	90% of motor threshold	20	–	Daily for five consecutive days	7 days after modeling	Sham TENS	2–3% isoflurane
Inoue et al., 2003	Rat	Sprague Dawley	–	Male	250–280	CCI	1	The back of the rat extended vigorously and the head moved backward	16.7	Ipsilateral	Daily for five consecutive days	7th–11th day after modeling	None	Sodium pentobarbital 40 mg/kg i.p. or 2% halothane
Lin et al., 2015	Rat	Sprague Dawley	–	Male	200–250	CCI	100	80% of motor threshold	20	Ipsilateral	Daily for 13 consecutive days	One day after modeling	None	2% isoflurane
Matsuo et al., 2014	Mouse	ICR/JCL	9	Male	39.6	SNI	100	Sub-motor threshold	30	Ipsilateral	Daily for seven consecutive days	1 and 2 weeks after modeling	Sham TENS	Anesthesia
Somers and Clemente, 1998	Rat	Sprague Dawley	–	Male	150–165	CCI	100	80% of motor threshold	90 or 60	Ipsilateral	Daily for 14, 13, or 11 consecutive days	Immediately, 20–30 h, or 3 days after modeling	None	Halothane (4%, maintained at 0.2–0.5%)
Somers and Clemente, 2003	Rat	Sprague Dawley	–	Male	150–165	CCI	100	80% of motor threshold	90 on the first day and then 60	Ipsilateral	Daily for 12 consecutive days	Immediately after modeling	None	Halothane (4%, maintained at 0.2–0.5%)
Somers and Clemente, 2006	Rat	Sprague Dawley	–	Male	170–200	CCI	100	80% of motor threshold	60	Ipsilateral Contralateral	Daily for 12 consecutive days	Beginning on the day of modeling	None	Halothane (4%, maintained at 0.2–0.5%)
Somers and Clemente, 2009	Rat	Sprague Dawley	–	Male	150–175	CCI	100	80% of motor threshold	90 on the first day and then 60	Contralateral	Daily for 12 consecutive days	Beginning on the day of modeling	None	Halothane (4%, maintained at 0.2–0.5%)
Su et al., 2018	Rat	Sprague Dawley	–	Male	250–300	NCI	5 or 100	–	30	–	Daily for seven consecutive days	Immediately or 7 days after modeling	None	1% isoflurane

*CCI, chronic constriction injury; i.p., intraperitoneal; SNI, spared nerve injury; SNL, spinal nerve ligation; TENS, transcutaneous electrical nerve stimulation; NCI, nerve crush injury.*

As shown in Table 2, paw withdrawal threshold to a mechanical stimulus was used to measure neuropathic pain in 10 studies (Somers and Clemente, 1998, 2003, 2006, 2009; Nam et al., 2001; Vera-Portocarrero et al., 2013; Cho et al., 2014; Matsuo et al., 2014; Lin et al., 2015; Su et al., 2018), followed by thermal threshold was reported in eight studies (Somers and Clemente, 1998, 2003, 2006, 2009; Inoue et al., 2003; Cho et al., 2014; Matsuo et al., 2014; Su et al., 2018) and cold threshold in three studies (Nam et al., 2001; Vera-Portocarrero et al., 2013; Cho et al., 2014). With regard to the timing of outcome measurement, the short-term effects were reported in three studies using a single session of TENS (Nam et al., 2001; Vera-Portocarrero et al., 2013; Cho et al., 2014) and nine studies employing repetitive TENS (Somers and Clemente, 1998, 2003, 2006, 2009; Vera-Portocarrero et al., 2013; Matsuo et al., 2014; Lin et al., 2015), while two studies (Inoue et al., 2003; Su et al., 2018) explored the long-term effects of repetitive TENS (up to 14 and 28 days after TENS, respectively). None studies reported mortality and adverse events related to TENS treatment.

**TABLE 2:** Characteristics of outcome evaluations, *K* = 12.

Times of TENS	Study	Mechanical threshold		Thermal threshold		Cold threshold		Timing of measurement	Adverse events
		Method	Relief of pain compared to control group	Method	Relief of pain compared to control group	Method	Relief of pain compared to control group		
Single session									
	Cho et al., 2014	von Frey Filaments	↑	Infrared generator	↑	Acetone	↑	Baseline and 30, 60, 90, 120, 180, and 240 h, and 1 day after TENS	–
	Nam et al., 2001	von Frey Filaments	↑	–	–	Ice	↔	30 min before, and at 30 min, 1, 2, 3, and 4 h after TENS	–
	Vera-Portocarrero et al., 2013*	von Frey Filaments	↔	–	–	Acetone	Unclear	Baseline and before and after TENS for five consecutive days	–
Multiple sessions									
	Inoue et al., 2003	–	–	Radiant heat	↔	–	–	Just before TENS, 7 days after modeling, and 1, 3, 7, and 14 days after the final TENS	–
	Vera-Portocarrero et al., 2013	von Frey Filaments	↔	–	–	Acetone	Unclear	Baseline and before and after electrical stimulation for five consecutive days	–
	Lin et al., 2015	von Frey Filaments	↑	–	–	–	–	Baseline and 3, 7, 11, and 14 days after modeling	–
	Matsuo et al., 2014	Analgesia-meter	Early↑ 1-week↔ 2-week↔	Radiant heat	Early↑ 1-week↔ 2-week↔	–	–	Before and every after modeling	–
	Somers and Clemente, 1998	Calibrate Semmes-Weinstein monofilaments	Immediately TENS ↔ 1-day TENS↔ 3-day TENS↑	Radiant heat	Immediately TENS↑ 1-day TENS↑ 3-day TENS↔	–	–	Baseline and then 2, 7, 12, and 14 days after modeling.	–
	Somers and Clemente, 2003	Calibrate Semmes-Weinstein monofilaments	↔	Radiant heat	↔	–	–	Baseline and 12 days after modeling	–
	Somers and Clemente, 2006	Calibrate Semmes-Weinstein monofilaments	High-frequency contralateral TENS↑ High-frequency ipsilateral TENS↔ Low-frequency contralateral TENS↔ Low-frequency ipsilateral TENS↔	Radiant heat	High-frequency contralateral TENS↔ High-frequency ipsilateral TENS↔ Low-frequency contralateral TENS↑ Low-frequency ipsilateral TENS↔	–	–	Baseline and 12 days after modeling	–
	Somers and Clemente, 2009	Calibrate Semmes-Weinstein monofilaments	High-frequency contralateral TENS↑ Low-frequency contralateral TENS↔	Radiant heat	High-frequency contralateral TENS↔ Low-frequency contralateral TENS↔	–	–	Baseline and 12 days after modeling	–
	Su et al., 2018	von Frey Filaments	High-frequency immediately TENS↓ High-frequency 1-week TENS↔ Low-frequency immediately TENS↔ Low-frequency 1-week TENS↔	Hot-plate test	–	–	–	Baseline, 7, 14, 21, and 28 days	–

*Vera-Portocarrero et al. (2013) provided the data on the efficacy of TENS following a single intervention.

*TENS, transcutaneous electrical nerve stimulation; ↔, no statistically significant improvement; ↑: significantly improvement; ↓: significantly deterioration.*

### Quality Assessment

According to the SYRCLE’s risk of bias tool for animal studies, the overall quality of existing literature was low due to the unclear risk of bias that existed in most of the studies (Supplementary Material 3). In terms of allocation sequence, we judged six out of the 11 included studies (Somers and Clemente, 1998, 2003, 2009; Inoue et al., 2003; Matsuo et al., 2014; Su et al., 2018) did not adequately describe the allocation sequence and the remaining five studies (Nam et al., 2001; Somers and Clemente, 2006; Vera-Portocarrero et al., 2013; Cho et al., 2014; Lin et al., 2015) did not report the random component in this process, and thereby we classified them as unclear risk for selection bias. Similarly, all the studies were rated as unclear risk for selection and detection biases because all the studies did not report or did not adequately report the method of allocation sequence concealment and whether the outcomes were measured randomly. Seven studies (Nam et al., 2001; Inoue et al., 2003; Vera-Portocarrero et al., 2013; Cho et al., 2014; Matsuo et al., 2014; Lin et al., 2015; Su et al., 2018) showed the intervention and control groups were comparable at baseline and thereby were rated as low risk of bias, while the other four studies (Somers and Clemente, 1998, 2003, 2006, 2009) did not report the similarity of groups and therefore were classified as unclear risk of bias. The majority of studies reported that animals were housed identically throughout the experiment and were rated as low risk of bias, except one study (Somers and Clemente, 1998) in which the housing conditions were omitted to report were classified as unclear risk of bias. In contrast, all of the included studies except one study (Vera-Portocarrero et al., 2013) were rated as unclear risk for performance bias due to the omitting of reporting whether TENS was performed in a blinded fashion. Only three studies (Nam et al., 2001; Lin et al., 2015; Su et al., 2018) did report the assessor was blinded, one study (Vera-Portocarrero et al., 2013) explicitly described the outcomes assessment did not conduct blindly, and the others did not mention this issue. Regarding attrition bias, six studies were classified as unclear risk of bias, of which three (Somers and Clemente, 1998, 2003; Su et al., 2018) did not report the total number of animals, two (Nam et al., 2001; Inoue et al., 2003) did not explain the reason why the animals dropped out, and the remaining one (Vera-Portocarrero et al., 2013) did not specify the number of animals per group. All of the included studies were rated as low risk for report bias, except one study (Su et al., 2018) that did not report the results of thermal threshold as planned was classified as high risk for report bias. Two studies were classified as unclear risk for other bias due to the lack of TENS intensity (Su et al., 2018) and the potential conflict of interest (Vera-Portocarrero et al., 2013). Four studies provided data related to the intra-rater reliability of the outcome measurement (Somers and Clemente, 1998, 2003, 2006, 2009).

### Meta-Analysis 1: The Effect of a Single Session of Transcutaneous Electrical Nerve Stimulation on Neuropathic Pain

Three studies (Nam et al., 2001; Vera-Portocarrero et al., 2013; Cho et al., 2014) assessed the effect of a single session of TENS on neuropathic pain and all of them provided data that could be included in the meta-analysis for the neuropathic pain. Of these, the mechanical threshold data of one study (Cho et al., 2014) could not be extracted from the presented figure, thereby the thermal threshold result was used as an alternative.

**FIGURE 2:**
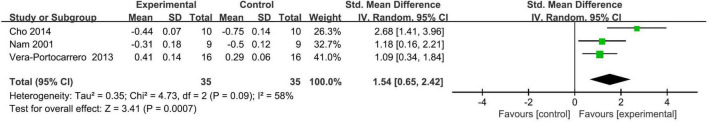
Forest plot of the effect of a single TENS on neuropathic pain versus controls.

Meta-analysis showed that a single session of TENS has a positive short-term effect in alleviating neuropathic pain relative to comparators (SMD: 1.54; 95% CI: 0.65, 2.42; *p* = 0.0007; *I*^2^ = 58%; Figure 2). The overall finding that a single session of TENS significantly alleviated neuropathic pain did not differ after omitting any single study of the included studies (Supplementary Material 4A). The number of included studies was too small to conduct reliable analyses of predefined subgroup and publication bias.

### Meta-Analysis 2: The Effect of Repetitive Transcutaneous Electrical Nerve Stimulation on Neuropathic Pain

Nine studies (Somers and Clemente, 1998, 2003, 2006, 2009; Inoue et al., 2003; Vera-Portocarrero et al., 2013; Matsuo et al., 2014; Lin et al., 2015; Su et al., 2018) with 18 comparisons measured the efficacy of repetitive TENS for neuropathic pain. All of these studies provided available data of mechanical threshold, except one study measured neuropathic pain by the paw withdrawal threshold to a thermal stimulus and therefore the thermal threshold result was included as an alternative.

**FIGURE 3:**
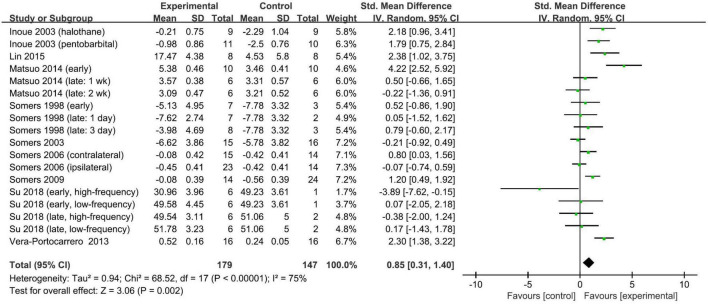
Forest plot of the effect of repetitive TENS on neuropathic pain versus controls.

Overall, repetitive TENS was shown to have a positive effect in alleviating neuropathic pain. Repetitive TENS groups significantly ameliorated neuropathic pain relative to comparators (SMD: 0.85; 95% CI: 0.31, 1.40; *p* = 0.002; *I*^2^ = 75%; Figure 3).

**FIGURE 4:**
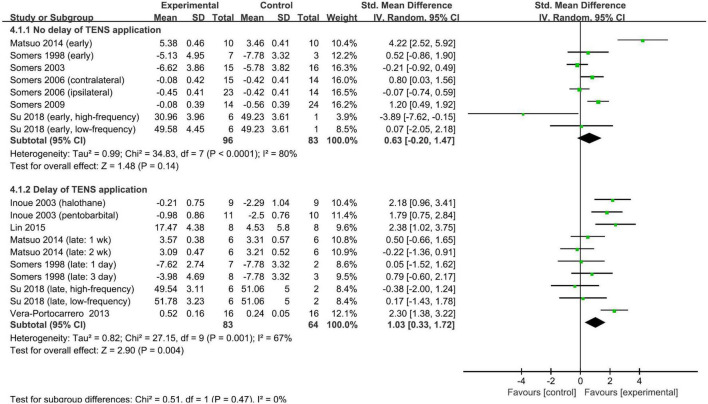
Subgroup analysis of the effect of repetitive TENS based on the timing of TENS application.

The timing of the application of TENS relative to pain modeling was used to stratify the subgroups. However, the pooled SMDs did not differ significantly (test for subgroup difference, *p* = 0.47): the pooled SMD was 0.63 (95% CI: −0.20, 1.47; *p* = 0.14; *I*^2^ = 80%; Figure 4) for studies with TENS commencing on the day of modeling and 1.03 (95% CI: 0.33, 1.72; *p* = 0.004; *I*^2^ = 67%; Figure 4) for studies with the delay of the TENS application. The other predefined stratified analyses were not conducted due to the insufficient number of studies. The overall finding that repetitive TENS significantly ameliorated neuropathic pain persisted in the leave-one-out sensitivity analyses (Supplementary Material 4B). Funnel plots were not performed to analyze the publication bias because the number of included studies was small.

## Discussion

For the first time, the present meta-analysis included 11 studies that specifically investigated the efficacy of TENS for neuropathic pain in rodent models, and overall, current data suggest that both a single session of TENS and repetitive TENS treatment might temporarily alleviate neuropathic pain in rodent models of neuropathic pain. Of note, the efficacy of repetitive TENS in ameliorating neuropathic pain is not varied by the timing of the application of TENS (no delay or delay). And the results persisted in the leave-one-out sensitivity analyses, which may in part prove the robustness of this meta-analysis. This meta-analysis provides a proof of concept for the application of TENS in pain caused by nerve injury. However, the high risk of bias in the included studies shows that care must be taken when interpreting these findings, and the small number of studies leaves many unsolvable knowledge gaps. Finally, evidence from the review does not support the generalization of the findings of the present meta-analysis to female animals and the long-term nociceptive effect of TENS remains unclear.

The disadvantages of clinical trials are that independent variables and confounders (Volz et al., 2012), including the timing of the application of TENS, cannot be controlled, leading to the insufficient understanding of whether TENS can be utilized in acute or chronic phases of pain caused by nerve injury. To this end, we systematically reviewed animal studies examining the nociceptive effects of TENS and stratified the included studies in terms of the timing of the application of TENS. Data from the present meta-analysis support both the early and delayed application of repetitive TENS, which might provide an appropriate time frame for TENS practice. Specifically, repetitive TENS has the potential to alleviate neuropathic pain in both acute and relatively chronic phases.

The frequency of TENS may be another vital factor that needs to be taken into account, as preclinical studies suggested that high-frequency and low-frequency TENS might exert an analgesic effect through different mechanisms. Studies showed that high-frequency TENS may be mediated *via* δ-opioid receptor class, while low-frequency may work through μ-opioid receptor class and therefore its effects may be limited in people using opioids, as opioids act through the μ-opioid receptor (Leonard et al., 2010; Sluka et al., 2013; Hendawy and Abuelnaga, 2020). To account for the importance of this, we planned to undertake a subgroup analysis based on frequency. However, studies comparing high-frequency and low-frequency TENS are insufficient to conduct further analysis. Usually, in low-frequency TENS settings, the TENS unit delivers low-frequency stimulus at a high stimulus intensity, which is close to the tolerance limit of the individual (Pal et al., 2020). Therefore, the low-frequency TENS is inevitably uncomfortable and is often considered for those who do not respond to high-frequency TENS (Pal et al., 2020). Taken together, it is not surprising most studies utilized high-frequency as an intervention.

Intensity is another crucial factor in maximizing the TENS effect, and it is suggested to maintain the level of intensity throughout TENS procedures by titrating to produce a strong, non-painful sensation (Bjordal et al., 2003; Moran et al., 2011; Sluka et al., 2013; Gibson et al., 2017). A study has shown that the intensity of TENS should be titrated upward to avoid habituation during TENS treatment (Pantaleão et al., 2011). However, all of the included studies did not report on the adjustment of the intensity of TENS during the experiment. Therefore, further animal studies investigating the efficacy of TENS should consider the adjustment of TENS intensity to optimize the efficacy of TENS.

Electrode placement may affect the effect of TENS in ameliorating neuropathic pain. It is found that high-frequency TENS applied contralaterally to the nerve injury better relieves the pain intensity (Somers and Clemente, 2006, 2009), whereas the lack of studies investigating the effect of contralateral TENS prevented the further analysis. Future studies should further confirm the efficacy of contralateral TENS and investigate whether contralateral TENS is frequency-dependent. Once its efficacy is proven, it will provide a useful reference for clinical use in the future.

Anesthesia administrated during TENS procedures requires consideration when investigating the efficacy of TENS as studies showed that anesthetics have properties of increasing (Antognini and Schwartz, 1993; Kingery et al., 2002) or reducing (Drasner, 2001) pain threshold. Furthermore, anesthetized animals during the intervention procedure cannot well mimic the clinical practice of TENS, since humans are commonly kept awake when receiving TENS therapy. Surface electrodes are commonly used in clinical practice; however, it is hard to maintain the placement of stimulation during experiment procedures using this type of electrodes (Chen et al., 2001). Further studies may consider the utilization of implanted electrodes or the development of alternative approaches that might eliminate these technical limitations.

According to the site of injury or disease, neuropathic pain can be classified as peripheral or central pain. Of the included studies, however, all of them used peripheral nerve injury pain models, including CCI, SNI, SNL, and NCI, resulting in whether the findings of the meta-analysis can be generalized to central neuropathic pain (e.g., central post-stroke pain and SCI-induced neuropathic pain) remaining unknown. There is an urgent need to investigate the effect of TENS on central neuropathic pain in future studies.

Sex is also a key factor for pain as both rodent and human studies reported sex differences in the physiologic and anatomical properties related to pain, including the expression and binding of mu-opioid receptor, morphine metabolism, the activation of the immune system, and the descending antinociceptive circuit (Fullerton et al., 2018). However, all of the included studies solely employed male rodents. Consideration must be given to further studies to explore whether the efficacy of TENS is varied by the sex of participants.

### Limitations and Strengths

Some limitations inevitably exist in the present meta-analysis. A study limitation is that there might have been several significant heterogeneities in the included studies, such as frequency, duration, and timing of TENS treatment and pain model. For meta-analytic aims, we had to merge these confounding factors in a meta-analysis and, although stratified the studies in terms of the timing of the application of TENS, other factors were analyzed simultaneously due to the insufficient number of included studies. Therefore, a random-effects model that considers this anticipated heterogeneity was utilized in the present meta-analysis. However, to avoid drawing wrong conclusions and thereby gain the most accurate, reliable, and reasonable findings, stratified analyses exploring the influence of the heterogeneity were conducted only if there were three or more comparable studies in a certain index. Another limitation is that the sample sizes of included studies are relatively low, which might restrict the statistical power (Hooijmans et al., 2014a; Huang et al., 2021; Velzen et al., 2021). However, The Principles of Humane Experimental Technique, also known as the “3 Rs,” called for every effort to reduce to a minimum the number of animals used in experiments (Flecknell, 2002), as such we would not overcriticize this issue. And fortunately, in meta-analyses of animal studies, it is suggested to focus on the effects direction rather than effect size itself, mainly due to the inevitable heterogeneity (Hooijmans et al., 2014a; Huang et al., 2021). Besides, some included studies did not mention the baseline data of intervention and control groups, which means we had to assess whether different groups were comparable and how this factor may influence the findings. Lastly, the majority of included studies did not or did not adequately describe the information regarding randomization, concealment, blinding, etc., possibly affecting the reliability of the analysis.

Despite these limitations, the present meta-analysis has some strengths. To obtain the most specific results, we included the largest number and most relevant animal studies published hitherto according to the rigorous criteria for inclusion and exclusion, which may be one of the strengths of the present meta-analysis. In addition, to obtain the most reasonable results and thereby help the future animal research design, we utilized the SYRCLE’s risk of bias tool for animal studies to evaluate the quality of current studies. Besides, meta-analyses of animal studies can explore the influence of the heterogeneity (Hooijmans et al., 2014a; Huang et al., 2021; Velzen et al., 2021), an important finding of this meta-analysis is that TENS was not a timing-dependent intervention, which may expand the applicability of TENS to some extent.

### Implications for Future Research

Future studies should therefore improve the quality of reporting, such as adequately describing the process of randomization, concealment, and binding and providing the sample size calculation of animals, to increase our confidence in estimating the efficacy of TENS. In addition, there is a need for a high quality of study design in future studies. The intensity of TENS should be titrated during the TENS procedures to produce a prespecified stimulation. In terms of outcome measurement, we would strongly recommend all related outcomes be accurately reported at baseline and all measurement times. This would greatly help the future evaluation of effect, including immediately, short-term, and long-term. Valid evaluations of the function should also be a critical reportable outcome in future studies. Safety data and adverse events should also be routinely monitored and reported as secondary outcomes, to explore how an increased stimulation dose of TENS can be reached. Anesthesia-free TENS is needed to develop to simulate the awake state that is maintained in clinical practice. Besides, female animals and central neuropathic pain models used in TENS studies would significantly help us learn more knowledge about the efficacy of TENS in these domains. Lastly, particular attention to study-level moderators and publication bias may augment the ability of research using animal models of neuropathic pain to optimize the efficacy of TENS for neuropathic pain and to know more about the mechanism underlying TENS treatment.

## Conclusion

The importance of this meta-analysis lies in the demonstration that, for TENS, both a single session and multiple sessions of applications lead to temporarily ameliorating the pain intensity in animal models of neuropathic pain, of which, repetitive TENS treatments are capable of alleviating neuropathic pain in both acute and relatively chronic phases. However, the direct extrapolation of the animal data to clinical practice is tenuous due to methodological limitations. Particular focus on the quality of TENS study design and reporting may increase the possibility of animal studies to predict the analgesic effect of TENS in humans, thus avoiding more potentially unsuccessful clinical trials, learning more about its therapeutic mechanism, and helping more people with neuropathic pain.

## Data Availability Statement

The original contributions presented in the study are included in the article/Supplementary Material, further inquiries can be directed to the corresponding author.

## Author Contributions

JH and YQ conceived and designed the study. JH, CY, and TW developed the search strategy. JH, ZZ, and YQ screened abstracts and full text reports. JH, YC, and YQ extracted outcomes. JH and KZ interpretation of the data. JH and CY wrote the manuscript. All authors contributed to the article and approved the submitted version.

## Conflict of Interest

The authors declare that the research was conducted in the absence of any commercial or financial relationships that could be construed as a potential conflict of interest.

## Publisher’s Note

All claims expressed in this article are solely those of the authors and do not necessarily represent those of their affiliated organizations, or those of the publisher, the editors and the reviewers. Any product that may be evaluated in this article, or claim that may be made by its manufacturer, is not guaranteed or endorsed by the publisher.
