# Supplementary material for: Transcutaneous Electrical Nerve Stimulation in Rodent Models of Neuropathic Pain: A Meta-Analysis
Source: Front Neurosci. 2022 Jan 31;16:831413. doi: 10.3389/fnins.2022.831413 (PMC8841820; doi:10.3389/fnins.2022.831413)
Supplement: Supplementary file 1 [file Table_1.DOCX]

**Supplementary material**

**Supplementary Material 1:** PRISMA checklist

**Supplementary Material 2:** Literature search strategy used in the current review.

**Supplementary Material 3:** Quality assessment of included studies.

**Supplementary Material 4:** Results of leave-one-out sensitivity analysis investigating the efficacy of TENS for neuropathic pain.

**Supplementary Material 1.** PRISMA checklist.

| **Section and Topic** | **Item #** | **Checklist item** | **Location where item is reported** |
| --- | --- | --- | --- |
| **TITLE** | | |  |
| Title | 1 | Identify the report as a systematic review. | Page 1 |
| **ABSTRACT** | | |  |
| Abstract | 2 | See the PRISMA 2020 for Abstracts checklist. | Page 1-2 |
| **INTRODUCTION** | | |  |
| Rationale | 3 | Describe the rationale for the review in the context of existing knowledge. | Page 2-3 |
| Objectives | 4 | Provide an explicit statement of the objective(s) or question(s) the review addresses. | Page 3 |
| **METHODS** | | |  |
| Eligibility criteria | 5 | Specify the inclusion and exclusion criteria for the review and how studies were grouped for the syntheses. | Page 3 |
| Information sources | 6 | Specify all databases, registers, websites, organisations, reference lists and other sources searched or consulted to identify studies. Specify the date when each source was last searched or consulted. | Page 3 |
| Search strategy | 7 | Present the full search strategies for all databases, registers and websites, including any filters and limits used. | Supplementary Material 2 |
| Selection process | 8 | Specify the methods used to decide whether a study met the inclusion criteria of the review, including how many reviewers screened each record and each report retrieved, whether they worked independently, and if applicable, details of automation tools used in the process. | Page 3 |
| Data collection process | 9 | Specify the methods used to collect data from reports, including how many reviewers collected data from each report, whether they worked independently, any processes for obtaining or confirming data from study investigators, and if applicable, details of automation tools used in the process. | Page 4 |
| Data items | 10a | List and define all outcomes for which data were sought. Specify whether all results that were compatible with each outcome domain in each study were sought (e.g. for all measures, time points, analyses), and if not, the methods used to decide which results to collect. | Page 4 |
|  | 10b | List and define all other variables for which data were sought (e.g. participant and intervention characteristics, funding sources). Describe any assumptions made about any missing or unclear information. | Page 4 |
| Study risk of bias assessment | 11 | Specify the methods used to assess risk of bias in the included studies, including details of the tool(s) used, how many reviewers assessed each study and whether they worked independently, and if applicable, details of automation tools used in the process. | Page 4 |
| Effect measures | 12 | Specify for each outcome the effect measure(s) (e.g. risk ratio, mean difference) used in the synthesis or presentation of results. | Page 4 |
| Synthesis methods | 13a | Describe the processes used to decide which studies were eligible for each synthesis (e.g. tabulating the study intervention characteristics and comparing against the planned groups for each synthesis (item #5)). | Page 4 |
|  | 13b | Describe any methods required to prepare the data for presentation or synthesis, such as handling of missing summary statistics, or data conversions. | Page 4 |
|  | 13c | Describe any methods used to tabulate or visually display results of individual studies and syntheses. | Page 4 |
|  | 13d | Describe any methods used to synthesize results and provide a rationale for the choice(s). If meta-analysis was performed, describe the model(s), method(s) to identify the presence and extent of statistical heterogeneity, and software package(s) used. | Page 4 |
|  | 13e | Describe any methods used to explore possible causes of heterogeneity among study results (e.g. subgroup analysis, meta-regression). | Page 4 |
|  | 13f | Describe any sensitivity analyses conducted to assess robustness of the synthesized results. | Page |
| Reporting bias assessment | 14 | Describe any methods used to assess risk of bias due to missing results in a synthesis (arising from reporting biases). | Page 4 |
| Certainty assessment | 15 | Describe any methods used to assess certainty (or confidence) in the body of evidence for an outcome. | Page 4 |
| **RESULTS** | | |  |
| Study selection | 16a | Describe the results of the search and selection process, from the number of records identified in the search to the number of studies included in the review, ideally using a flow diagram. | Page 4-5  Figure 1 |
|  | 16b | Cite studies that might appear to meet the inclusion criteria, but which were excluded, and explain why they were excluded. | Figure 1 |
| Study characteristics | 17 | Cite each included study and present its characteristics. | Page 5 -6  Table 1-2 |
| Risk of bias in studies | 18 | Present assessments of risk of bias for each included study. | Page 6  Supplementary Material 3 |
| Results of individual studies | 19 | For all outcomes, present, for each study: (a) summary statistics for each group (where appropriate) and (b) an effect estimate and its precision (e.g. confidence/credible interval), ideally using structured tables or plots. | Page 6-7  Figure 2-4 |
| Results of syntheses | 20a | For each synthesis, briefly summarise the characteristics and risk of bias among contributing studies. | Page 6-7  Table 1-2 |
|  | 20b | Present results of all statistical syntheses conducted. If meta-analysis was done, present for each the summary estimate and its precision (e.g. confidence/credible interval) and measures of statistical heterogeneity. If comparing groups, describe the direction of the effect. | Page 6-7  Figure 2-4 |
|  | 20c | Present results of all investigations of possible causes of heterogeneity among study results. | Figure 4  Supplementary Material 4 |
|  | 20d | Present results of all sensitivity analyses conducted to assess the robustness of the synthesized results. | Page 6-7  Supplementary Material 4 |
| Reporting biases | 21 | Present assessments of risk of bias due to missing results (arising from reporting biases) for each synthesis assessed. | Page 6 Supplementary Material 3 |
| Certainty of evidence | 22 | Present assessments of certainty (or confidence) in the body of evidence for each outcome assessed. | Page 6-7  Supplementary Material 4 |
| **DISCUSSION** | | |  |
| Discussion | 23a | Provide a general interpretation of the results in the context of other evidence. | Page 7 |
|  | 23b | Discuss any limitations of the evidence included in the review. | Page 9 |
|  | 23c | Discuss any limitations of the review processes used. | Page 9 |
|  | 23d | Discuss implications of the results for practice, policy, and future research. | Page 9 |
| **OTHER INFORMATION** | | |  |
| Registration and protocol | 24a | Provide registration information for the review, including register name and registration number, or state that the review was not registered. | Page 3 |
|  | 24b | Indicate where the review protocol can be accessed, or state that a protocol was not prepared. | Page 3 |
|  | 24c | Describe and explain any amendments to information provided at registration or in the protocol. | N/A |
| Support | 25 | Describe sources of financial or non-financial support for the review, and the role of the funders or sponsors in the review. | Page 10 |
| Competing interests | 26 | Declare any competing interests of review authors. | Page 10 |
| Availability of data, code and other materials | 27 | Report which of the following are publicly available and where they can be found: template data collection forms; data extracted from included studies; data used for all analyses; analytic code; any other materials used in the review. | Page 3 |

**Supplementary Material 2.** Literature search strategy used in the current review.

Database: Cochrane Library (to November 2021)

- #1 MeSH descriptor: [Pain] explode all trees
- # 2 (pain* or ache or aches or neuropathic pain* or neuralgic pain* or neuralgia* or neurodynia* or allodynia* or hyperalgesia* or chronic pain* or nerve injur* or nerve damag* or nerve compress* or nerve trauma* or nerve lesion*):ti,ab,kw
- # 3 #2 OR #1
- # 4 MeSH descriptor: [Transcutaneous Electric Nerve Stimulation] explode all trees
- # 5 (transcutaneous electrical nerve stimulation or transcutaneous electric* nerve stimulation or transcutaneous nerve stimulation or electric* nerve stimulation electric* nerve therap* or electroanalgesia or TENS or TNS or ENS):ti,ab,kw
- # 6 #5 OR #4
- # 7 MeSH descriptor: [Muridae] explode all trees
- # 8 (Muridae OR mouse OR mice OR rat OR rats OR rodent* OR murine):ti,ab,kw
- #9 #7 OR #8
- #10 #9 AND #6 AND #3

Database: EMBASE (to November 2021)

- #1 exp pain/
- #2 (pain* or ache or aches or neuropathic pain* or neuralgic pain* or neuralgia* or neurodynia* or allodynia* or hyperalgesia* or chronic pain* or nerve injur* or nerve damag* or nerve compress* or nerve trauma* or nerve lesion*).ab,ti.
- #3 1 or 2
- #4 exp transcutaneous electrical nerve stimulation/
- #5 (transcutaneous electrical nerve stimulation or transcutaneous electric* nerve stimulation or transcutaneous nerve stimulation or electric* nerve stimulation electric* nerve therap* or electroanalgesia or TENS or TNS or ENS).ab,ti.
- #6 4 or 5
- #7 exp Muridae/
- #8 (Muridae OR mouse OR mice OR rat OR rats OR rodent* OR murine).ab,ti.
- #9 7 or 8
- #9 3 and 6 and 9

Database: MEDLINE (via PubMed, to November 2021)

- #1 (pain[MeSH Terms]) OR (pain*[Title/Abstract] OR ache[Title/Abstract] OR aches[Title/Abstract] OR neuropathic pain*[Title/Abstract] OR neuralgic pain*[Title/Abstract] OR neuralgia*[Title/Abstract] OR neurodynia*[Title/Abstract] OR allodynia*[Title/Abstract] OR hyperalgesia*[Title/Abstract] OR chronic pain*[Title/Abstract] OR nerve injur*[Title/Abstract] OR nerve damag*[Title/Abstract] OR nerve compress*[Title/Abstract] OR nerve trauma*[Title/Abstract] OR nerve lesion*[Title/Abstract])
- #2 (Transcutaneous Electrical Nerve Stimulation[MeSH Terms]) OR (transcutaneous electrical nerve stimulation[Title/Abstract] OR transcutaneous electric* nerve stimulation[Title/Abstract] OR transcutaneous nerve stimulation[Title/Abstract] OR electric* nerve stimulation electric* nerve therap*[Title/Abstract] OR electroanalgesia[Title/Abstract] OR TENS[Title/Abstract] OR TNS[Title/Abstract] OR ENS[Title/Abstract])
- #3 (Muridae[MeSH Terms]) OR (Muridae[Title/Abstract] OR mouse[Title/Abstract] OR mice[Title/Abstract] OR rat[Title/Abstract] OR rats[Title/Abstract] OR rodent*[Title/Abstract] OR murine[Title/Abstract])
- #4 #1 AND #2 AND #3

Database: Web of science (to November 2021)

- #1 TS=pain
- # 2 TI=(pain* or ache or aches or neuropathic pain* or neuralgic pain* or neuralgia* or neurodynia* or allodynia* or hyperalgesia* or chronic pain* or nerve injur* or nerve damag* or nerve compress* or nerve trauma* or nerve lesion*) OR KP=(pain* or ache or aches or neuropathic pain* or neuralgic pain* or neuralgia* or neurodynia* or allodynia* or hyperalgesia* or chronic pain* or nerve injur* or nerve damag* or nerve compress* or nerve trauma* or nerve lesion*)
- # 3 #2 OR #1
- # 4 TS= transcutaneous electrical nerve stimulation
- # 5 TI=(transcutaneous electrical nerve stimulation or transcutaneous electric* nerve stimulation or transcutaneous nerve stimulation or electric* nerve stimulation electric* nerve therap* or electroanalgesia or TENS or TNS or ENS) OR KP=(transcutaneous electrical nerve stimulation or transcutaneous electric* nerve stimulation or transcutaneous nerve stimulation or electric* nerve stimulation electric* nerve therap* or electroanalgesia or TENS or TNS or ENS)
- # 6 #5 OR #4
- # 7 TS=Muridae
- # 8 TI=(Muridae OR mouse OR mice OR rat OR rats OR rodent* OR murine) OR KP=(Muridae OR mouse OR mice OR rat OR rats OR rodent* OR murine)
- #9 #7 OR #8
- #10 #9 AND #6 AND #3

**Supplementary Material 3.** Quality assessment of included studies.

| Item | Bias | Cho et al., 2014 | Nam et al., 2001 | Vera-Portocarrero et al., 2013 | Inoue et al., 2003 | Lin et al., 2015 | Matsuo et al., 2014 | Somers et al., 1998 | Somers et al., 2003 | Somers et al., 2006 | Somers et al., 2009 | Su et al., 2018 |
| --- | --- | --- | --- | --- | --- | --- | --- | --- | --- | --- | --- | --- |
| 1 | Sequence generation (Selection bias) | U | U | U | U | U | U | U | U | U | U | U |
| 2 | Baseline characteristics (Selection bias) | Y | Y | Y | Y | Y | Y | U | U | U | U | Y |
| 3 | Allocation concealment (Selection bias) | U | U | U | U | U | U | U | U | U | U | U |
| 4 | Random housing (Performance bias) | Y | Y | Y | Y | Y | Y | U | U | Y | Y | Y |
| 5 | Blinding (Performance bias) | U | U | Y | U | U | U | U | U | U | U | U |
| 6 | Random outcome assessment (Detection bias) | U | U | U | U | U | U | U | U | U | U | U |
| 7 | Blinding (Detection bias) | U | Y | N | U | Y | U | U | U | U | U | Y |
| 8 | Incomplete outcome data (Attrition bias) | Y | U | U | U | Y | Y | U | U | Y | Y | U |
| 9 | Selective outcome reporting (Reporting bias) | Y | Y | Y | Y | Y | Y | Y | Y | Y | Y | N |
| 10 | Other sources of bias | Y | Y | U | Y | Y | Y | Y | Y | Y | Y | U |

Y: low risk of bias; N, high risk of bias; U, unclear risk of bias

**Supplementary Material 4.** Results of leave-one-out sensitivity analysis investigating the efficacy of TENS for neuropathic pain.

1. Results of leave-one-out sensitivity analysis investigating the efficacy of single TENS for neuropathic pain (Figure 2).

| Study excluded | Overall analysis | | |
| --- | --- | --- | --- |
|  | SMD | *I*^2^ (%) | *p* |
| Cho et al. 2014 | 1.12 [0.52, 1.72] | 0 | 0.00003 |
| Nam et al. 2001 | 1.80 [0.24, 3.35] | 78 | 0.02 |
| Vera-Portocarrero el al. 2013 | 1.88 [0.42, 3.35] | 69 | 0.01 |

1. Leave-one-out sensitivity analysis when stratifying studies according to the timing of TENS (Figure 4).

| Subgroup | Study excluded | Subgroup analysis | | | Overall analysis | | | Test for subgroup difference | |
| --- | --- | --- | --- | --- | --- | --- | --- | --- | --- |
|  |  | SMD | *I*^2^ (%) | *p* | SMD | *I*^2^ (%) | *p* | *p* | *I*^2^ (%) |
| No delay of TENS application | Matsuo et al. 2014 (early) | 0.30 [-0.32, 0.92] | 62 | 0.34 | 0.71 [0.21, 1.21] | 70 | 0.005 | 0.13 | 57.5 |
|  | Somers et al. 1998 (early) | 0.64 [-0.29, 1.58] | 83 | 0.18 | 0.87 [0.30, 1.44] | 77 | 0.003 | 0.52 | 0 |
|  | Somers et al. 2003 | 0.79 [-0.18, 1.76] | 80 | 0.11 | 0.94 [0.37, 1.50] | 73 | 0.001 | 0.69 | 0 |
|  | Somers et al. 2006 (contralateral) | 0.59 [-0.43, 1.61] | 82 | 0.25 | 0.85 [0.26, 1.45] | 77 | 0.005 | 0.49 | 0 |
|  | Somers et al. 2006 (ipsilateral) | 0.76 [-0.24, 1.76] | 81 | 0.14 | 0.92 [0.35, 1.49] | 74 | 0.002 | 0.66 | 0 |
|  | Somers et al. 2009 | 0.52 [-0.45, 1.49] | 80 | 0.29 | 0.82 [0.23, 1.41] | 76 | 0.007 | 0.40 | 0 |
|  | Su et al. 2018 (early, high-frequency) | 0.81 [0.00, 1.61] | 80 | 0.05 | 0.93 [0.40, 1.46] | 74 | 0.0006 | 0.68 | 0 |
|  | Su et al. 2018 (early, low-frequency) | 0.68 [-0.21, 1.58] | 83 | 0.14 | 0.88 [0.32, 1.44] | 76 | 0.002 | 0.55 | 0 |
| Delay of TENS application | Inoue el al. 2003 (halothane) | 0.90 [0.16, 1.63] | 67 | 0.02 | 0.77 [0.22, 1.32] | 75 | 0.006 | 0.64 | 0 |
|  | Inoue el al. 2003 (pentobarbital) | 0.93 [0.15, 1.70] | 69 | 0.02 | 0.79 [0.22, 1.35] | 75 | 0.006 | 0.61 | 0 |
|  | Lin el al. 2015 | 0.89 [0.16, 1.61] | 67 | 0.02 | 0.77 [0.22, 1.32] | 75 | 0.006 | 0.65 | 0 |
|  | Matsuo el al. 2014 (late: 1 wk) | 1.09 [0.32, 1.85] | 69 | 0.005 | 0.87 [0.30, 1.45] | 77 | 0.003 | 0.43 | 0 |
|  | Matsuo el al. 2014 (late: 2 wk) | 1.20 [0.51, 1.88] | 61 | 0.0006 | 0.92 [0.36, 1.49] | 76 | 0.001 | 0.31 | 4.0 |
|  | Somers el al. 1998 (late: 1 day) | 1.12 [0.39, 1.85] | 68 | 0.003 | 0.89 [0.33, 1.46] | 76 | 0.002 | 0.39 | 0 |
|  | Somers el al. 1998 (late: 3 day) | 1.05 [0.28, 1.81] | 70 | 0.007 | 0.85 [0.28, 1.43] | 77 | 0.003 | 0.47 | 0 |
|  | Su el al. 2018 (late, high-frequency) | 1.16 [0.46, 1.87] | 66 | 0.001 | 0.91 [0.35, 1.47] | 76 | 0.001 | 0.34 | 0 |
|  | Su el al. 2018 (late, low-frequency) | 1.11 [0.37, 1.84] | 69 | 0.003 | 0.88 [0.32, 1.45] | 76 | 0.002 | 0.40 | 0 |
|  | Vera-Portocarrero el al. 2013 | 0.86 [0.16, 1.56] | 60 | 0.02 | 0.75 [0.21, 1.29] | 72 | 0.006 | 0.68 | 0 |

SMD: standardized mean difference.
